# Anisotropic local covariance matrices for spatial blind source separation

**DOI:** 10.1007/s10182-025-00529-2

**Published:** 2025-06-10

**Authors:** Christoph Muehlmann, Claudia Cappello, Sandra De Iaco, Klaus Nordhausen

**Affiliations:** 1 Institute of Statistics and Mathematical Methods in Economics, Vienna University of Technology, Wiedner Hauptstrasse 8-10, 1040 Vienna, Austria; 2 DSE - Section of Mathematics and Statistics, University of Salento, Via per Monteroni, 73100 Lecce, Italy; 3 Department of Mathematics and Statistics, University of Helsinki, P.O. Box 68, 00014 Helsinki, Finland; 4 Department of Mathematics and Statistics, University of Jyväskylä, P.O. Box 35, 40014 Jyvaskyla, Finland

**Keywords:** Spatial statistics, Covariance function, Isotropy

## Abstract

This paper aims to introduce a novel approach to spatial blind source separation (SBSS) that addresses the limitations of existing methods. Current SBSS techniques rely on the joint diagonalization of multiple local covariance functions, all of which assume isotropy. To overcome this constraint, anisotropic local covariance matrices that relax the isotropy assumption are proposed. A simulation study and an application on real-world data demonstrate the performance improvement obtained by incorporating these anisotropic covariance matrices into the SBSS framework and highlight the potential of this new approach for more accurate and flexible source separation in spatial data analysis.

## Introduction

Modeling multivariate geostatistical data poses significant challenges, as dependencies between variables and locations must be considered. This is typically accomplished by modeling the multivariate (cross-) covariance function $\textbf{C}(\textbf{x}_{\textbf{s}_i},\textbf{x}_{\textbf{s}_j})$, where a *p*-variate vector $\textbf{x}$ is observed at locations $\textbf{s}_1,\ldots , \textbf{s}_n \in {\mathcal{S}} \subset \mathbb{R}^d$ and ${\mathcal{S}}$ denotes the spatial domain. In most applications $d=2$, which is also our main interest in the subsequent analysis. Genton and Kleiber (2015) highlight the difficulties in specifying and estimating valid models for $\textbf{C}(\textbf{x}_{\textbf{s}_i},\textbf{x}_{\textbf{s}_j})$, noting that convenient yet strong assumptions are often made to facilitate modeling.

One such assumption is that the covariance function is isotropic, meaning it depends only on the distance $||\textbf{h}||$ between the involved locations $\textbf{s}_i$ and $\textbf{s}_j$, i.e.,

$$\textbf{C}(\textbf{x}_{\textbf{s}_i},\textbf{x}_{\textbf{s}_j}) := \textbf{C}(||\textbf{s}_i- \textbf{s}_j ||) := \textbf{C}(||\textbf{h} ||).$$

This results in a covariance function that is second-order stationary and invariant under rotations. However, the appropriateness of these assumptions is debatable, and various proposals in the literature attempt to relax one or both assumptions (Journel and Huijbregts 1976; Sampson and Guttorp 1992; Cressie 1993; Paciorek and Schervish 2006; Allard et al. 2016). Approaches that do not assume rotational invariance are referred to as anisotropic approaches. Sherman (2011) explains the severe consequences of erroneously assuming isotropy, particularly in the context of prediction.

Many multivariate spatial methods directly parametrize the covariance function $\textbf{C}(\textbf{x}_{\textbf{s}_i},\textbf{x}_{\textbf{s}_j})$ and estimate its parameters accordingly. However, an alternative approach is provided by spatial blind source separation (SBSS), which operates within a semi-parametric latent variable framework. Instead of modeling the multivariate field as a whole, SBSS seeks to decompose it into latent univariate fields that can then be modeled individually, ideally simplifying the choice of covariance functions.

SBSS was first introduced by Nordhausen et al. (2015) and has since undergone several methodological advancements (Bachoc et al. 2020; Muehlmann et al. 2024a; Piccolotto et al. 2022; Sipilä et al. 2024a). The core idea of SBSS is to use spatially weighted local scatter matrices to identify a transformation that separates the original multivariate process into uncorrelated or independent latent fields. These fields are then assumed to exhibit different spatial dependence structures, which can be analyzed independently. Traditionally, SBSS has been developed and applied under the assumption of isotropy, meaning that latent fields are estimated assuming isotropic covariance functions.

More recently, efforts have been made to extend SBSS beyond the stationary isotropic assumption and allow for more flexible spatial structures. This includes approaches that relax the assumption of stationarity (Muehlmann et al. 2024b, 2022; Bachoc et al. 2024; Sipilä et al. 2024b).

Applications of SBSS have so far mainly focused on geochemical data (Nordhausen et al. 2015; Muehlmann et al. 2021; Bachoc et al. 2024; Sipilä et al. 2024b), where latent components often exhibit different spatial structures due to varying geological processes.

In this paper, an approach to relax the isotropy assumption in SBSS, allowing for the identification of latent fields that exhibit anisotropic spatial dependencies, has been proposed.

After a brief review SBSS in Sect. 2, then the suggested method for accounting for anisotropy in Sect. 3 has been presented. Finally, the effects of this approach have been demonstrated in a simulation study in Sect. 4 and in an example on real data in Sect. 5.

## Spatial blind source separation

Spatial blind source separation (SBSS) is a semiparametric approach that assumes the observable *p*-variate random field $\textbf{x}(\textbf{s})$ can be expressed as

$$\textbf{x}(\textbf{s})= \textbf{A} \textbf{z}(\textbf{s})+ \textbf{b},$$

where the full-rank $p \times p$ matrix $\textbf{A}$ is the mixing matrix, the *p*-variate vector $\textbf{b}$ represents the mean vector, and $\textbf{z}(\textbf{s})= \left( z_1,\ldots ,z_p\right) ^\top$ is a *p*-variate latent random field with the following properties:

*SBSS*1 :  $E(\textbf{z}(\textbf{s})) = \textbf{0}$ and $\mathop {\text{Cov}}\nolimits (\textbf{z}(\textbf{s})) = \textbf{I}_p$ for all $\textbf{s} \in {\mathcal{S}}$.
*SBSS*2 :  The components of $\textbf{z}(\textbf{s})$ are mutually independent.
*SBSS*3 :  Let $\textbf{h} = \textbf{s}_i-\textbf{s}_j$, then $\textbf{C}(\textbf{z}_{\textbf{s}_i},\textbf{z}_{\textbf{s}_j}) = \textbf{D}_{\textbf{h}}$, where $\textbf{D}_{\textbf{h}}$ is a diagonal matrix whose diagonal elements depend only on $\textbf{h}$.

Thus, formally SBSS assumes second-order stationarity up to this point, and its goal is to estimate a $p \times p$ unmixing matrix $\textbf{W}$ such that $\textbf{W} (\textbf{x}(\textbf{s})- \textbf{b})$ has independent components. The underlying idea is that, due to the independence of the components, *p* univariate models can be fitted, which is much simpler than constructing a *p*-variate model directly, see for example Muehlmann et al. (2021).

Nordhausen et al. (2015), Bachoc et al. (2020) obtain the unmixing matrix by jointly diagonalizing the covariance matrix and $K \ge 1$ local covariance matrices, defined as:

1 $$\begin{aligned} \textbf{M}_f(\textbf{x}(\textbf{s})) = \frac{1}{n} \sum _{i,j = 1}^n f(\textbf{s}_i - \textbf{s}_j) \mathop {\text{E}}\nolimits \Bigl [ [\textbf{x}(\textbf{s}_i) - \mathop {\text{E}}\nolimits (\textbf{x}(\textbf{s}_i))] [\textbf{x}(\textbf{s}_j)- \mathbf {\mathop {\text{E}}\nolimits }(\textbf{x}(\textbf{s}_j)) ]^\top \Bigr ], \end{aligned}$$

where $f:\mathbb{R}^ d \rightarrow \mathbb{R}$ represents a spatial kernel function. Moreover, Nordhausen et al. (2015) suggested ball kernel functions $f_b(\textbf{h}) = I(|| \textbf{h} || \le r)$, while Bachoc et al. (2020) additionally considered ring and Gauss kernel functions. The ring kernel function is given by $f_r(\textbf{h}) = I(r_{1} < || \textbf{h} || \le r_2)$, and the Gauss kernel function $f_g(\textbf{h}) = {\text{exp}}(-0.5 (\Phi ^{-1}(0.95) || \textbf{h} || / r)^2)$, where $\Phi ^{-1}(0.95)$ is the $95\%$ quantile of a standard normal distribution, can be regarded as a smooth version of the ball kernel function.

An unmixing matrix $\textbf{W}$ can be defined as the $p \times p$ matrix that maximizes

2 $$\begin{aligned} \sum _{k=1}^K||\mathop {\text{diag}}\nolimits \left( \textbf{W}\textbf{M}_{f_k}\textbf{W}^\top \right) ||^2 \end{aligned}$$

subject to the constraint $\textbf{W}\mathbf {\mathop {\text{Cov}}\nolimits }(\textbf{x}(\textbf{s})) \textbf{W}^\top = \textbf{I}_p$ and $||\cdot ||$ is the Frobenius norm. Here, $\mathop {\text{diag}}\nolimits (\textbf{A})$ is a diagonal matrix with the same diagonal elements as in $\textbf{A}$, and $\textbf{M}_{f_k}$ represents *K* local covariance matrices with distinct kernels $f_1,\ldots ,f_K$. Depending on the values of *K* and the specific kernels used, the methods exhibit various properties that enable different algorithms but necessitate distinct additional assumptions. In general, these assumptions require that the covariance functions of $z_i$ must all be different. For more details, see Nordhausen et al. (2015), Bachoc et al. (2020). Note also that Bachoc et al. (2020, 2024) considered (1) with additional normalization factors which make asymptotic considerations easier but yield the same unmixing matrix.

It is crucial to note that all local covariance matrices constructed in this manner implicitly assume isotropy, with no directional information involved; they depend solely on the distance between observations. In the following section, a new approach to constructing local covariance matrices that addresses this limitation will be introduced.

## Anisotropic local covariance

Isotropic models represent obviously a subset of the class of second-order stationary covariance functions; however, this can represent a restrictive assumption. The new anisotropic local covariance matrix $\textbf{M}_{\varvec{\alpha }, f}$ is defined as $\textbf{M}_{\varvec{\alpha },f}(\textbf{x})$

3 $$\begin{aligned} = n ^ {-1} \sum _{i,j=1}^n f(\textbf{h}_{ij}) I(\textbf{h}_{ij} \angle \textbf{e}_{\alpha _1} \in [0, \alpha _2 ]) \mathop {\text{E}}\nolimits \Bigl ( (\textbf{x}(\textbf{s}_i) - \mathop {\text{E}}\nolimits (\textbf{x}(\textbf{s}_i))) (\textbf{x}(\textbf{s}_j)- \mathbf {\mathop {\text{E}}\nolimits }(\textbf{x}(\textbf{s}_j)) )^\top \Bigr ). \end{aligned}$$

**Fig. 1:**
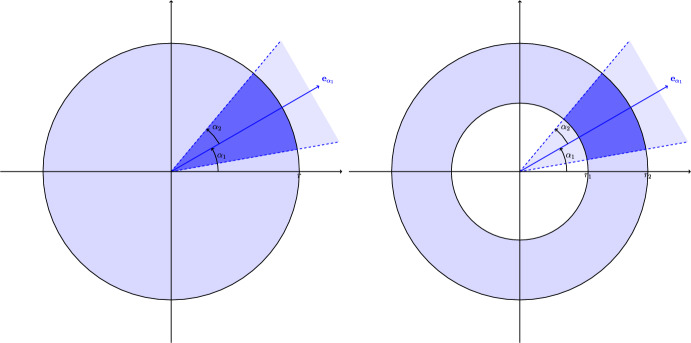
Illustration of the ball (left panel) and ring (right panel) spatial kernel functions together with the indicator function that incorporates the anisotropic aspect. The region where vectors within the angle $\alpha _2$ from $\textbf{e}_{\alpha _1}$ satisfy the condition is shaded dark blue

Here, $\textbf{h}_{ij} = \textbf{s}_i - \textbf{s}_j$, $I(\cdot )$ is the indicator function, $\varvec{\alpha }=(\alpha _1, \alpha _2)^\top$ and $\angle$ denotes the angle between two vectors, $\textbf{e}_{\alpha _1} = (\cos {\alpha _1}, \sin {\alpha _1})^\top$ with $\alpha _1 \in [0, 2 \pi ]$, $\alpha _2 \in [0, \pi / 2]$. $f: \mathbb{R}^d \rightarrow \mathbb{R}$ is a spatial kernel function which can be still chosen out of selection mentioned above and according to the discussions by Nordhausen et al. (2015), Bachoc et al. (2020). To account for anisotropy, the novel addition is the indicator function that is added and which excludes coordinate pairs where the angle of the separation vector $\textbf{h}_{ij}$ relative to the x-axis does not lie between $\alpha _1$ and $\alpha _2$. The effect of the indicator function is illustrated in Fig. 1 for a spatial ball and a spatial ring kernel function. Therefore, an anisotropic local covariance matrix does not only take the distance between locations into consideration but also includes directional information.

Basically the idea is then to estimate the unmixing matrix in the same way as in (2) by now jointly diagonalizing the covariance matrix and $K \ge 1$ different anisotropic local covariance matrices. However, in the formulation of the optimization problem in (2), it is necessary that all matrices to be diagonalized are symmetric, which does not necessarily hold for all $\textbf{M}_{\varvec{\alpha },f}(\textbf{x})$. This can be nevertheless simply overcome by symmetrizing $\textbf{M}_{\varvec{\alpha },f}(\textbf{x})$, i.e.,

4 $$\begin{aligned} \textbf{M}_{\varvec{\alpha },f}^S(\textbf{x}) \rightarrow 2^{-1} (\textbf{M}_{\varvec{\alpha },f}(\textbf{x}) + \textbf{M}_{\varvec{\alpha },f}(\textbf{x})^\top ). \end{aligned}$$

Therefore, for anisotropic random fields which follow the SBSS model, the estimation of the unmixing matrix $\textbf{W}$ has to be properly set. In particular, the proposal is to estimate the unmixing matrix $\textbf{W}$ by modifying (2) and maximizing

5 $$\begin{aligned} \sum _{k=1}^K||\mathop {\text{diag}}\nolimits \left( \textbf{W}\textbf{M}_{(\varvec{\alpha },f)_k}^S\textbf{W}^\top \right) ||^2 \end{aligned}$$

under the constraint $\textbf{W}\mathbf {\mathop {\text{Cov}}\nolimits }(\textbf{x}(\textbf{s})) \textbf{W}^\top = \textbf{I}_p$ and $||\cdot ||$ is the Frobenius norm.

That an anisotropic unmixing matrix based on (5) is indeed better than the isotropic one from (2) in the case of anisotropic data is then demonstrated in the following simulation study (Sect. 4) and through an application of a real-word data (Sect. 5).

## Simulations

The simulation design follows a three-step procedure: (i) Different three-variate random fields following a SBSS model with known mixing matrix and spatial covariance structure are simulated on a set of coordinates, (ii) different SBSS estimators are applied on the simulated dataset to obtain estimates for the unmixing matrix and (iii) the estimated unmixing matrices and the known mixing matrix are compared by a performance measure. These three steps are repeated 2000 times, and the average performance measure for each setting is presented. Below the details for each step and the results are given. The statistical computations for the simulation study were performed using the R environment (version 4.0.3; R Core Team 2023), with the aid of the RandomFields (Schlather et al. 2015), JADE (Miettinen et al. 2017), and SpatialBSS (Muehlmann et al. 2025) packages. The latter provides implementations for both isotropic and anisotropic SBSS.

***Sample Locations & Random Field Models*** Given the spatial domains of the form $\mathcal{S}= [0,n] \times [0,n]$ where $n = 10, 20, 30, 40, 50, 60, 70$, the set of sample locations for each domain is the integer grid $\mathcal{C}= \mathcal{S}\cap \mathbb{Z}^2$. In the following, the domains have been denoted loosely by $[0,n]^2$.

Regarding the random field, four different settings have been considered. Each setting consists of three-variate Gaussian random fields following the SBSS model defined above where the mixing matrix equals the identity $\mathbf {\textbf{A}}= \textbf{I}_3$ and $\textbf{b} = \textbf{0}$. The spatial covariance structure for the latent components, depending on the separation vector $\textbf{h}= \textbf{s}- \textbf{s}'$ for any two points $\textbf{s}$ and $\textbf{s}'$, is given by

6 $$\begin{aligned} C(\textbf{h}) = \frac{\sigma _1 ^ 2}{2 ^ {\nu - 1} \Gamma (\nu )} \left( \frac{\Vert \textbf{B}_1 \textbf{h}\Vert }{\phi } \right) ^ \nu K_\nu \left( \frac{\Vert \textbf{B}_1 \textbf{h}\Vert }{\phi } \right) + \sigma _2^2 I(\textbf{B}_2 \textbf{h}= \textbf{0}). \end{aligned}$$

The first part of the covariance functions is the well-known stationary Mátern covariance (Guttorp and Gneiting 2006) with $\sigma _1^2 > 0$, $\nu > 0$ and $\phi > 0$ are the variance, shape and range parameter, $K_\nu$ is the modified Bessel function of second kind, and $\Gamma$ is the gamma function. The second part is an on-site variance term often denoted as nugget term with the variance parameter $\sigma _2^2 >= 0$. With this definition, geometric anisotropy can be included by the coordinate transformation given by the matrix $\textbf{B}_1$ defined as:

7 $$\begin{aligned} \textbf{B}_1 = \begin{pmatrix} 1 & 0 \\ 0 & 1 / r \end{pmatrix} \begin{pmatrix} \cos {\beta } & - \sin {\beta } \\ \sin {\beta } & \cos {\beta } \end{pmatrix}. \end{aligned}$$

Hence, the coordinates get rotated by the angle $\beta$ and then the length of the (new) second axis is reduced by the factor 1/*r* with $r>0$. Zonal anisotropy can be included by the nugget effect in conjunction with the matrix $\textbf{B}_2$, which is supposed to be a matrix of rank one defined by

8 $$\begin{aligned} \textbf{B}_2 = \textbf{b} \textbf{b}^\top . \end{aligned}$$

With this definition, a constant variance $\sigma _2^2$ is added to the model when a pair of sample locations has zero distance along the direction given by the vector $\textbf{b}$, or equivalently, a constant variance $\sigma _2^2$ is added to the direction perpendicular to $\textbf{b}$.

With this, the four considered settings can be specified as follows.

1. Isotropic setting with $\sigma ^2_1 = 1$, $\sigma ^2_2 = 0$, $\beta = 0$ and $r = 1$ for all three latent fields. The parameters $(\nu , \phi )$ equal (0.7, 1.0) for the first, (1.0, 1.5) for the second and (1.3, 2.0) for the third entry of the latent field.
2. Zonal anisotropic setting with $\sigma ^2_1 = 0.7$, $\sigma ^2_2 = 0.3$ for all three latent fields and $(\nu , \phi )$ as in Setting 1. The vectors $\textbf{b}$ equal $(1,1)^\top$ for the first, $(1, 0)^\top$ for the second and $(0, 1)^\top$ for the third entry of the latent field.
3. Geometric anisotropic setting with $\sigma ^2_1 = 1$, $\sigma ^2_2 = 0$ for all three latent fields and $(\nu , \phi )$ as in Setting 1. The parameters $(\beta , r)$ equal $(\pi / 8, 3)$ for the first, $(\pi / 3, 2)$ for the second and $(2 \pi / 3, 5)$ for the third entry of the latent field.
4. Zonal and geometric anisotropic setting with $\sigma ^2_1 = 0.7$, $\sigma ^2_2 = 0.3$ for all three latent fields, $(\nu , \phi )$ as in Setting 1, the vectors $\textbf{b}$ as in Setting 2 and $(\beta , r)$ as in Setting 3.

**Fig. 2:**
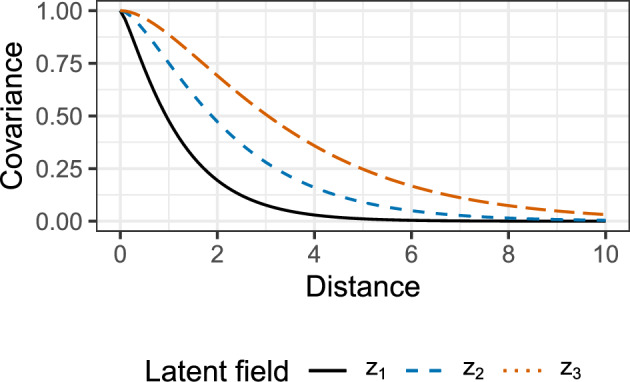
The covariance functions for the three latent components used in the isotropic Setting 1

Figure 2 depicts the spatial covariance functions of the three latent components in case of isotropy (Setting 1), and Table 1 summarizes the included form of anisotropy for each setting.

**Table 1:** Summary of the class of anisotropy for all considered simulation settings

Setting	Zonal	Geometric
1	−	−
2	+	−
3	−	+
4	+	+

***Estimators*** For each estimator, three ring kernels with parameters $(r_1, r_2) \in \{ (0,1), (1,2), (2,3)\}$ have been used, but different configurations of the angles $\varvec{\alpha }=(\alpha _1, \alpha _2)^\top$ for each of the three rings have also been considered. Hence, the difference in performance of the methods originates mostly from the chosen angles. Specifically, five different sets of angles, which are summarized in Table 2, have been introduced. Lastly, the unmixing matrix has been estimated by the fourth-order blind identification (FOBI) method (Cardoso 1989; Nordhausen and Virta 2019) which does not account for spatial dependence at all; thus, it acts as a reference method or “random guess.”

**Table 2:** Summary of the considered angles for the SBSS estimators

Name	$\varvec{\alpha }$	Total $\#$ kernels
Aniso.x	$(0, \pi / 4)$	3
Aniso.y	$(\pi / 2, \pi / 4)$	3
Aniso.1	$(\pi / 4, \pi / 4)$, $(3 \pi / 4, \pi / 4)$	6
Aniso.2	$(\pi / 4, \pi / 8)$, $(\pi / 2, \pi / 8)$, $(3 \pi / 4, \pi / 8)$	9
Iso	$(0, \pi / 2)$	3

**Fig. 3:**
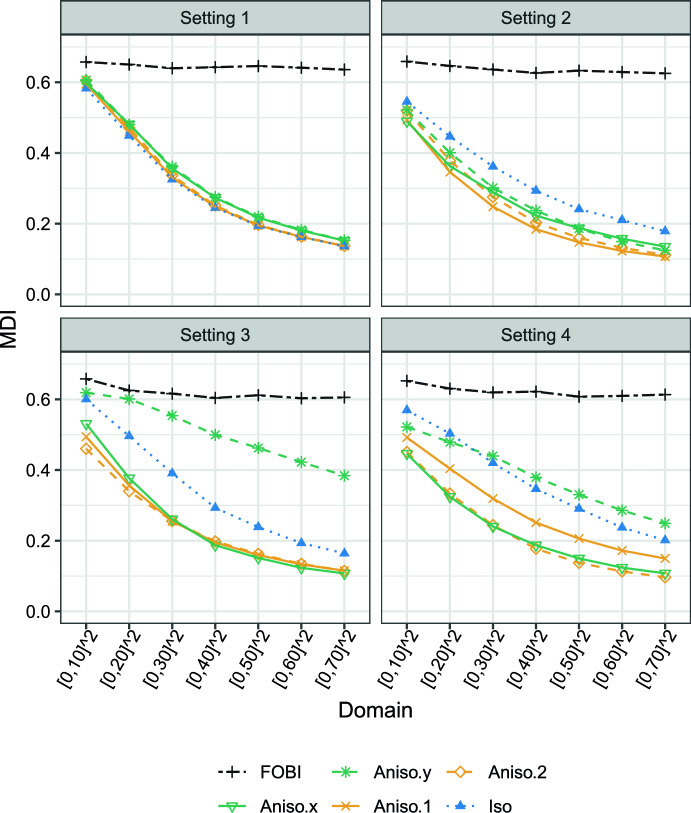
Mean MDI curves for various BSS methods based on 2000 simulation repetitions. Setting 1 represents the isotropic case, Setting 2 corresponds to the zonal anisotropic case, Setting 3 captures the geometric anisotropic case, and Setting 4 combines both zonal and geometric anisotropic features

***Performance Measure*** To measure the quality of an estimated unmixing matrix $\hat{\textbf{W}}$ based on the known mixing matrix $\textbf{A}$, one can exploit the fact that mixing and unmixing the signal should be the identity operation (up to the model indeterminacies of sign, order and scale) when the estimate is perfect, i.e., $\hat{\textbf{W}} \textbf{A} \approx \textbf{I}_p$. This consideration is used by the minimum distance index (MDI) (Ilmonen et al. 2010; Lietzen et al. 2020) which is constructed as

9 $$\begin{aligned} \text {MDI}(\hat{\textbf{W}}, \textbf{A}) = \frac{1}{\sqrt{p-1}} \inf _{\textbf{C} \in \mathcal{C}^p} \Vert \textbf{C} \hat{\textbf{W}} \textbf{A} - \textbf{I}_p \Vert _F. \end{aligned}$$

Here, $\mathcal{C}^p$ is the set of all $p \times p$ matrices with exactly one nonzero element on each row and column which accounts for the not identifiable order, scale and sign. The MDI takes values between zero and one where zero is a perfect estimation of the unmixing matrix.

***Results*** Figure 3 depicts mean MDI curves based on 2000 simulation repetitions for the combinations of all sample sizes, settings and estimators. From the simulation outcomes, the following qualitative conclusions can be drawn.

(i) In a fully isotropic setting, estimators with any choice of anisotropy deliver meaningful results.
(ii) Isotropic kernel settings might not be corrupted by sole zonal anisotropy; however, slightly better results are achieved by anisotropic kernels.
(iii) Accounting anisotropy, when the data have a geometric anisotropic nature, significantly improves the results. The considered directions must be cautious chosen; wrong direction kernel worsens the results significantly compared to isotropic kernels.
(iv) When in doubt of isotropy and corresponding directions, a save kernel options might be build upon kernels that use different directions which in total cover the whole circle.

In a space-time context, apart from a natural form of non-geometric anisotropy which is also justified by the non-comparability of the spatial and temporal dimensions of the domain, it is also reasonable that the spatial evolution is anisotropic; thus, the results of the spatio-temporal BSS in Muehlmann et al. (2023) can be further extended by considering the above-mentioned evidence.

## Case study

**Fig. 4:**
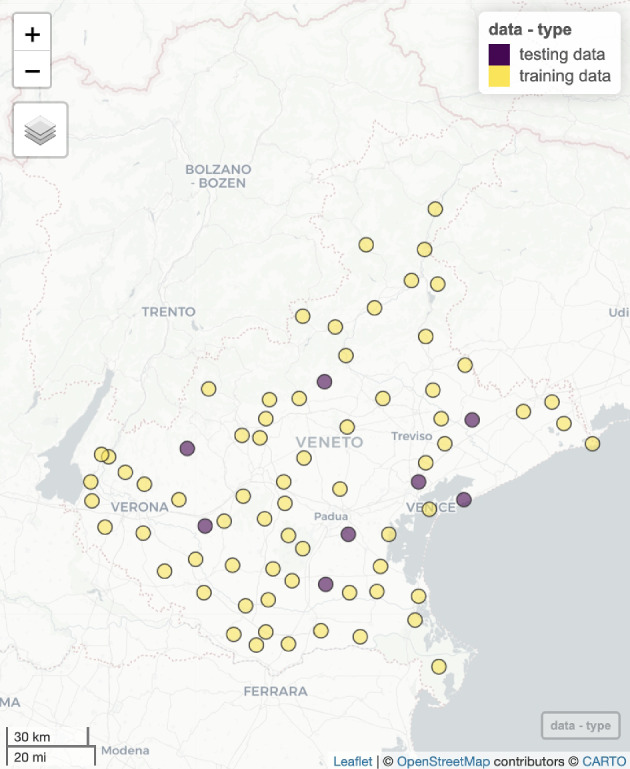
Location map of the 67 training stations and 8 testing stations, in the Veneto region

In this section, it is demonstrated how the proposed approach can be applied to real-world data. The dataset, available in the R package SpatialBSS (Muehlmann et al. 2025), contains the averages of various meteorological variables for week 28 of 2021. The data were collected by the Environmental Agency for Prevention and Protection of the Italian Veneto Region (https://www.arpa.veneto.it) from 75 monitoring stations across the region (Fig. 4). In particular, the following variables have been considered: evapotranspiration levels ($ET_0$, in *mm*) determined using the method proposed in (Hargreaves 1974), maximum and minimum air temperature ($T_M$ and $T_m$, respectively, in $^\circ C$), percentage of maximum and minimum relative humidity ($H_M$ and $H_m$, respectively) and precipitations (*P*) in *mm*. In the case study, a well-known Box-Cox-type transformation $ln(P + 1)$, denoted with *lP*, was employed; thus, taking into account the presence of zero values, before applying the log transformation to reduce skewness, the precipitation data have been shifted by 1.

The performance of the new anisotropic procedure has been evaluated through the leave-one-out cross-validation and the jack-knife estimations at 67 and 8 stations, respectively. Note that 8 spatial locations have been randomly selected from the available 75 sample points and have been used to define the testing dataset, leaving the remaining 67 monitoring stations in the training data. Then, a comparison between the results obtained using the anisotropic and isotropic approach has also been proposed.

**Fig. 5:**
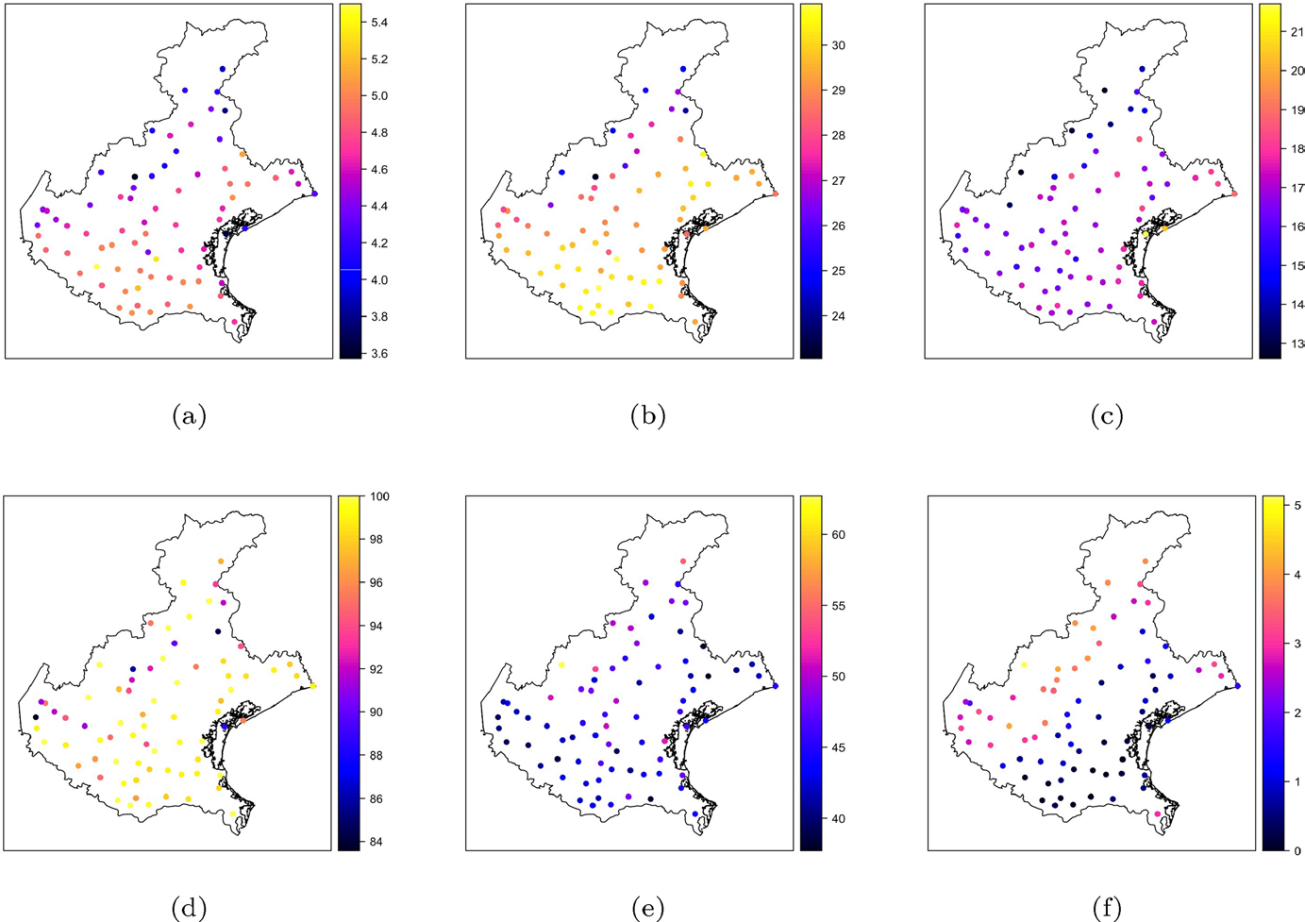
Color maps of **a** $ET_0$, **b** $T_M$, **c** $T_m$, **d** $H_M$, **e** $H_m$ and **f** *lP*

From the descriptive statistics and the color maps of the training data (Table 3, Fig. 5), it is evident that the scales differ; hence, standardized data with zero mean and unit variance have been considered in the SBSS procedure in order to facilitate the loadings interpretation. This data transformation does not affect the found latent components as the BSS methods are affine equivariant.

**Table 3:** Descriptive statistics of the meteorological variables

	Minimum	1 st Quartile	Median	Mean	3rd Quartile	Maximum	SD
$ET_{0}$	3.57	4.44	4.71	4.66	4.94	5.50	0.38
$T_{M}$	23.06	28.45	29.36	28.86	30.06	30.91	1.70
$T_{m}$	12.61	16.34	16.86	16.69	17.46	21.71	1.51
$H_{M}$	83.57	95.21	98.43	96.92	99.57	100.00	3.91
$H_{m}$	37.71	41.79	43.71	44.67	46.93	62.71	4.33
*lP*	0	0.47	1.39	1.80	3.05	5.14	1.47

After the exploratory data analysis, the variograms estimation has been performed for different directions and the presence of anisotropy has been detected.

Hence, the new defined local covariance matrices (Sect. 3) have been jointly diagonalized, and the weights, determined by the spatial kernels, have been properly chosen to account for the anisotropy. In particular, 12 ring kernels have been considered, with radii (0, 40) km, (40, 65) km, (65, 80) km and angles $0, \pi / 4, \pi / 2 \text{ and } 3\pi / 4$, which include all possible directions; moreover, the choice of the inner and outer radii has been supported by the evaluation of the descriptive statistics on the Euclidean distance among the spatial monitoring stations.

In Table 4 the estimated unmixing matrix is given. From the loadings, it is evident that:

- $z_1$ is mainly formed by the precipitations,
- $z_2$ is mostly driven by the difference between $T_m$ and $T_M$,
- $z_3$ is characterized by $ET_0$ and the discrepancy between $H_m$ and $H_M$,
- $z_4$ is described by $ET_0$ and the difference between $T_m$ and $T_M$,
- $z_5$ is formed by the discrepancy between $T_M$ and $T_m$, as well as $H_m$ and $H_M$, with also a negative contribution of $ET_0$
- $z_6$ is similar to $z_5$, except for the contribution of humidity.

**Table 4:** Unmixing matrix for the six anisotropic spatial latent random fields

	$ET_{0}$	$T_{M}$	$T_{m}$	$H_{M}$	$H_{m}$	*lP*
$z_{1}$	0.09	− 0.11	− 0.09	− 0.19	− 0.13	**0.90**
$z_{2}$	0.61	− **1.35**	**1.20**	0.41	0.71	− 0.03
$z_{3}$	**1.78**	− 0.04	0.01	− **0.76**	**1.21**	0.34
$z_{4}$	**1.25**	− **1.04**	**1.53**	0.30	− 0.13	0.85
$z_{5}$	− **1.84**	**3.30**	− **1.09**	− **1.08**	**0.73**	0.19
$z_{6}$	− **3.13**	**4.40**	− **1.81**	0.49	0.32	0.70

Bolded values highlight the variables used for interpretation

**Fig. 6:**
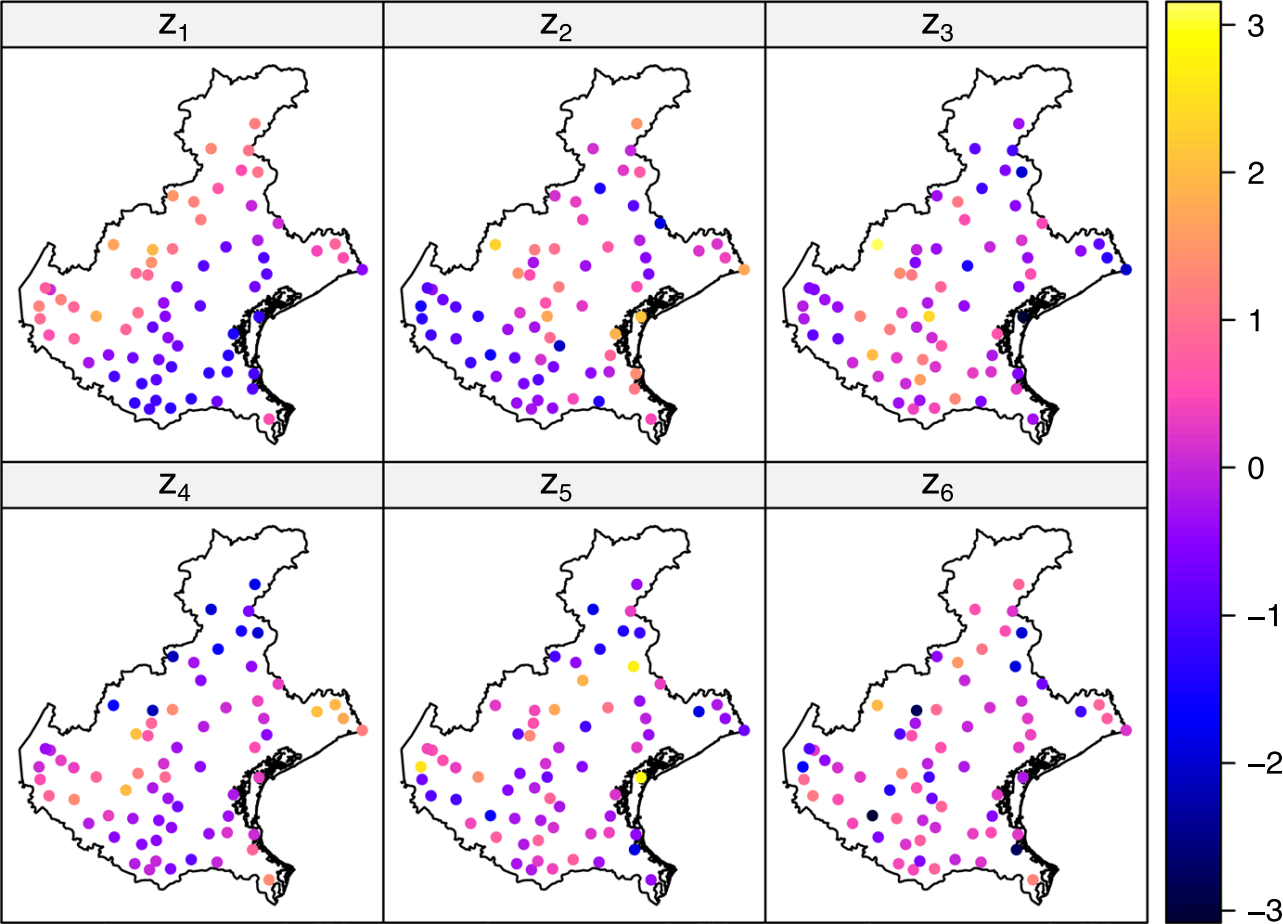
Anisotropic spatial latent random fields found by applying SBSS with 12 ring kernel functions

Figure 6 depicts the SBSS solution of the meteorological data using 12 ring kernel functions with parameters $(r_1, r_2) \in \{(0, 40), (40, 65), (65, 80)\} \, km$ and $(\alpha _1, \alpha _2) \in \{(0, \pi / 8), (\pi /4, \pi / 8), (\pi /2, \pi / 8), (3\pi /4, \pi / 8)\}$.

**Fig. 7:**
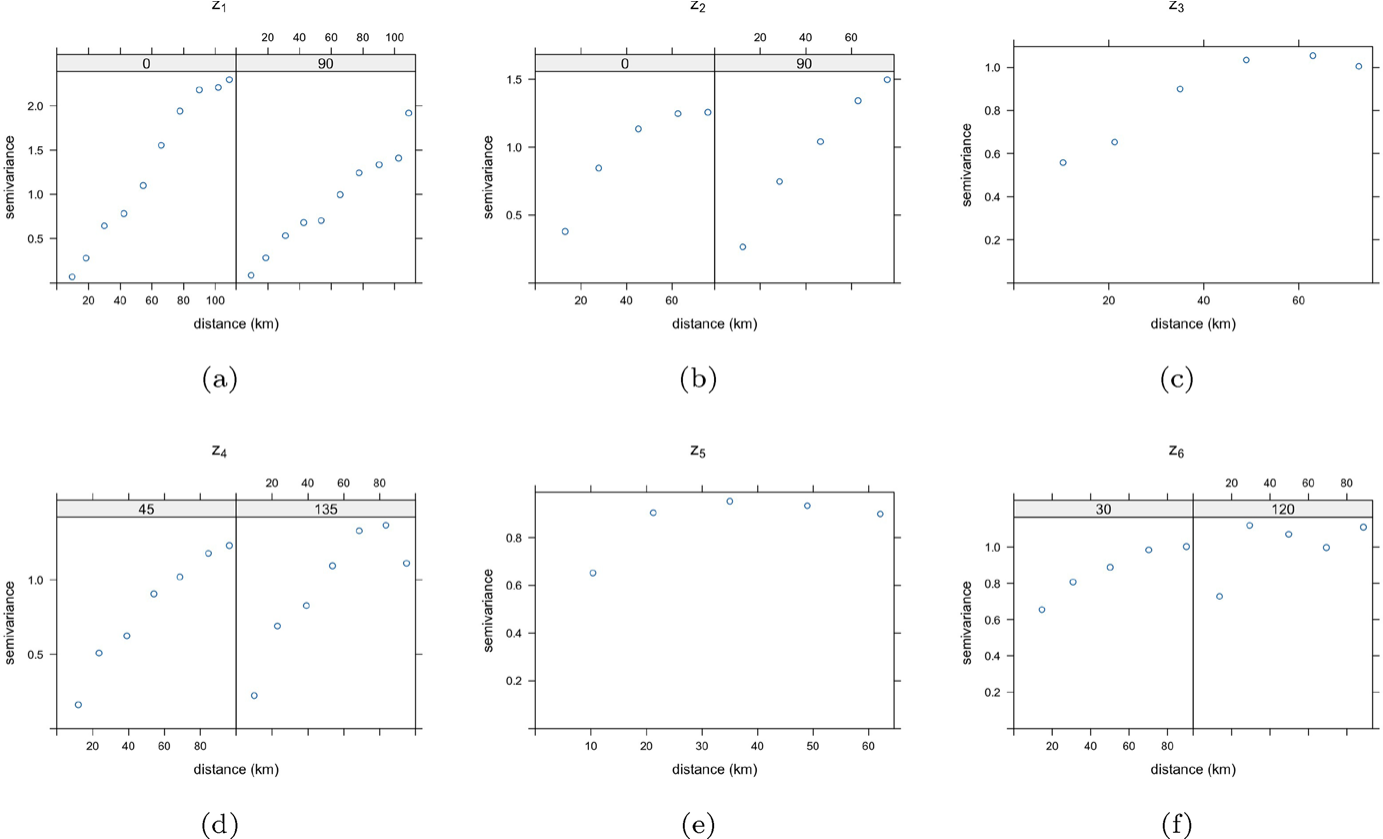
Omnidirectional and directional sample variograms for the six anisotropic spatial latent random fields

After the estimation of the uncorrelated latent components and of the unmixing matrix $\hat{\textbf{W}}$, each latent field $\hat{z}_i, \, i = 1, \dots , 6$, has been analyzed in a univariate manner, and the most appropriate variogram model has been fitted in order to predict each component at some unsampled spatial locations using the kriging method. To this aim, the omnidirectional and directional variograms (i.e., the ones associated with the angles $0^{\circ }$, $30^{\circ }$, $45^{\circ }$, $75^{\circ }$, $90^{\circ }$, $120^{\circ }$, $135^{\circ }$ and $165^{\circ }$) have been estimated for different spatial lags, where the number of lags has been properly defined according to the geometry of the spatial points over the domain. In Fig. 7 the omnidirectional variograms or the variograms along the directions of maximum and minimum continuity for each latent component are depicted.

From a graphical inspection of the sample variograms, the class of models (i.e., spherical, exponential or Gaussian) has been chosen and the corresponding parameters have been set, as detailed in Table 5. Note that 4 out of 6 components show an anisotropic behavior; hence, the anisotropy ratio (obtained as the ratio between the range in the direction of minimum continuity and the one along the direction of maximum continuity) has been specified. This characteristic revealed by the directional variograms might be influenced by the orientation of the locations toward the sea or the weather conditions in the analyzed week.

**Table 5:** Variogram model parameters for the six anisotropic spatial latent random fields

	Model	Nugget	Sill	Range (km)	Direction of max continuity	Anisotropy ratio
$z_1$	Gau	0.05	2.35	92.05	$90^{\circ }$	0.66
$z_2$	Sph	0	1.30	77.00	$90^{\circ }$	0.844
$z_3$	Gau	0.49	0.57	30.50	–	–
$z_4$	Sph	0	1.27	106.88	$45^{\circ }$	0.694
$z_5$	Exp	0	0.96	8.70	–	–
$z_6$	Exp	0.40	0.65	31.50	$30^{\circ }$	0.378

Before using the above-mentioned models to make predictions of the meteorological variables at 8 testing monitoring stations, the models’ adequacy has been checked, as described in the next section.

***Leave-one-out cross-validation validation model and comparison***

A leave-one-out cross-validation technique has been applied for each latent component, in order to assess the goodness of the fitted models detailed in Table 5 and the appropriateness of the anisotropic setting. The predicted values of the latent components have been multiplied by $\hat{\textbf{W}}$ to derive the variables of interest. Then among the error metrics proposed in the literature, the relative mean absolute error (rMAE) and the root average error (RAE) have been considered. In particular:

- the rMAE has been computed as the ratio between the sum of the absolute errors and the sum of the absolute value of the true measurements,
  10 $$\begin{aligned} \text{ rMAE }=\frac{\displaystyle {\sum _{i=1}^{n}\Big |\,\widehat{x}(\textbf{s}_i)-x(\textbf{s}_i)\Big |}}{\displaystyle \sum _{i=1}^{n} \Big |\, x(\textbf{s}_i)\,\Big |}, \end{aligned}$$
- the RAE has been computed as the square root of the ratio between the sum of the squared errors and the sum of the squared true values,
  11 $$\begin{aligned} \text{ RAE }=\left\{ \frac{\displaystyle {\sum _{i=1}^{n}\Big [\widehat{x}(\textbf{s}_i)-{x}(\textbf{s}_i)\Big ]^{2}}}{\displaystyle \sum _{i=1}^n x^{2}(\textbf{s}_i)}\right\} ^{0.5}. \end{aligned}$$

The correlation coefficient between the observed and the estimated values as well as the relative indexes (r = 0.998, rMAE = 0.039 and RAE = 0.049) has confirmed the adequacy of the variogram models fitted to the latent components as well as the satisfactory results of anisotropic SBSS in recovering the latent components themselves. However, it is important to consider that the leave-one-out cross-validation setup usually overestimates the prediction performance as the test and training sets are dependent, as highlighted in Otto et al. (2024).

The performance of the anisotropic SBSS has also been evaluated through a comparative analysis with respect to the results obtained from the traditional SBSS (in the isotropic case). Structural analysis has also been conducted for the independent components, retrieved from the isotropic SBSS, and the models provided in Table 6 have been fitted to the corresponding sample variograms.

**Table 6:** Variogram model parameters for the latent random fields estimated under the hypothesis of isotropy

	Model	Nugget	Sill	Range (km)
$z_1$	Gau	0.16	1.33	63.14
$z_2$	Gau	0.44	0.83	34.76
$z_3$	Exp	0.07	1.17	37.03
$z_4$	Gau	0.44	0.62	36.13
$z_5$	Sph	0	1.00	15.00
$z_6$	Exp	0	0.98	8.23

In Table 7, the values of the error indexes and the percentage relative variation ($\Delta$) between the indicator under the anisotropy assumption and the one under the isotropy hypothesis (relative to the latter) are given. Negative (positive) $\Delta$ values highlight an improvement (worsening) in predicting the variables under study, starting from the anisotropic SBSS latent fields and the corresponding mixing matrix, instead of the isotropic SBSS latent components and their mixing matrix. It is evident that the anisotropic approach shows better performance than the isotropic one, with a relative improvement up to 7.722% and 6.273% in terms of rMAE and RAE, respectively, and an overall $\Delta$ of 1.451% and 2.794%.

**Table 7:** Cross-validation results from anisotropic and isotropic approach

	$rMAE_{Anis}$	$rMAE_{Is}$	$\Delta$ (%)	$RAE_{Anis}$	$RAE_{Is}$	$\Delta$ (%)
$ET_0$	0.0398	0.0402	− 0.985	0.0588	0.0593	− 0.902
$T_M$	0.0226	0.0227	− 0.724	0.0340	0.0341	− 0.381
$T_m$	0.0498	0.0488	2.073	0.0682	0.0690	− 1.182
$H_M$	0.0301	0.0318	− 5.360	0.0408	0.0424	− 3.956
$H_m$	0.0556	0.0538	3.383	0.0756	0.0767	− 1.447
*lP*	0.2474	0.2681	− 7.722	0.2520	0.2689	− 6.273
Overall	0.0388	0.0394	− 1.451	0.0488	0.0502	− 2.794

***Spatial predictions and comparison***

In this stage of the analysis, the two SBSS approaches have been compared in terms of predictive performance, by estimating the analyzed variables at 8 testing monitoring stations.

In particular, the kriging estimates for all the latent components have been computed using the models detailed in Tables 5 and 6; then, the univariate estimates have been multiplied by the inverse of the unmixing matrix $\hat{\textbf{W}}$. Finally, these last results have been converted to the original scale of the data.

The predictive performance of the two SBSS approaches has been assessed by means of the error indexes, already used in the previous section, namely the rMAE and the RAE. For each index, a percentage relative variation $\Delta$ has been computed (Table 8).

**Table 8:** Statistics on the prediction errors from anisotropic and isotropic approach

	$rMAE_{Anis}$	$rMAE_{Is}$	$\Delta$ (%)	$RAE_{Anis}$	$RAE_{Is}$	$\Delta$ (%)
$ET_0$	0.0334	0.0370	− 9.502	0.0364	0.0391	− 6.946
$T_M$	0.0177	0.0183	− 3.026	0.0263	0.0269	− 2.092
$T_m$	0.0708	0.0650	8.864	0.0810	0.0777	4.315
$H_M$	0.0241	0.0270	− 10.600	0.0309	0.0336	− 8.173
$H_m$	0.0474	0.0513	− 7.554	0.0521	0.0566	− 7.958
*lP*	0.2726	0.3038	− 10.261	0.2443	0.2653	− 7.926
Global	0.0347	0.0369	− 6.093	0.0367	0.0394	− 6.777

The goodness of the anisotropic approach has also been confirmed when the observed variables are predicted at new spatial locations. Indeed, the deviation between estimates and true values is on average lower for proposed procedure with respect to the isotropic case, with values for the relative variation up to 10.600% and 8.173% in terms of rMAE and RAE, respectively, as well as a global improvement of the prediction errors of 6.093% and 6.777% in terms of the same error indexes.

The R code to reproduce the case study is available in the supplementary material.

## Discussion

Isotropy is a convenient yet restrictive assumption that has been implicitly employed in SBSS, as all the local covariance matrices involved did not incorporate any directional information. In this study, it is demonstrated how local covariance matrices can be modified to include directional information, which proved to be highly advantageous in the simulation study. The extension proposed here was formulated for $d=2$, where it effectively selects “slices” from a circle. Analogously, for $d=3$, the method could select “cones” from spheres. A pertinent question arises: Which slices or cones are important? Generally, subject-specific information should primarily guide this selection. However, as demonstrated in the case studies on simulated and real data, employing multiple slices can serve as a safeguard when in doubt.

It is worth noting that SBSS has recently been extended to the spatio-temporal domain by Muehlmann et al. (2023), and their approach also relied on similar local covariance matrices. The proposed method for incorporating directional information in the spatial component of the local space-time covariance function can be naturally applied in this context as well. However, as clarified, the impact may be less pronounced than in the purely spatial case presented here, as local space-time covariance matrices inherently exhibit anisotropy due to the distinct nature of space and time measures.
